# Inhibition of miMOMP-induced SASP to combat age-related disease

**DOI:** 10.3389/fragi.2025.1505063

**Published:** 2025-01-29

**Authors:** Xiaoli Liao, Zhennan Guo, Mouhai He, Yichun Zhang

**Affiliations:** ^1^ School of Medical Technology and Nursing, Hunan institute of traffic engineering, Hengyang, China; ^2^ Hengyang Xin Yahan Medical Beauty Clinic, Hengyang, China

**Keywords:** miMOMP, SASP (senescence-associated secretory phenotype), age-related disease, cGAS-STING, mtDNA

## Abstract

Cellular senescence, first described in 1961, was initially observed in normal human fibroblasts that ceased proliferating after a finite number of divisions in culture. This process is triggered by various stimuli, including oxidative stress, chromatin modifications and oncogene activation, characterized by irreversible cell-cycle arrest, resistance to apoptosis and the induction of a complex senescent associated secretory phenotype (SASP). Over the past decade, emerging evidence has linked cellular senescence to the aging process and a wide range of chronic age-related diseases. Consequently, research focused on targeting senescence to alleviate or delay age-related disease, referred to as senotherapy, has been conducted rapidly. Therefore, elucidating the mechanisms of cellular senescence is essential for providing practical strategies aimed at addressing this condition.

## Introduction

It is widely acknowledged that mitochondria play a crucial role in cellular apoptosis, a process distinct from cellular senescence (Calabrò et al., 2024). During apoptosis, widespread mitochondrial outer membrane permeabilization (MOMP), dependent on the formation of BAX/BAK macropores enabling the release of mitochondrial DNA (mtDNA) into the cytosol, leads to cell death (Bock and Tait, 2020). Apart from that, growing evidence has shown that mitochondria dysfunction is also a defining characteristic of cellular senescence and aging, while the best determinant of mitochondrial dysfunction in senescent cells and aging tissues is an increased MOMP (Correia-Melo et al., 2016; Miwa et al., 2022). However, the detailed role of mitochondrial and the underlying mechanism driving mitochondrial dysfunction in senescent cells remain unknown.

## miMOMP induces cellular senescence

In a recent issue of Nature, Victorelli et al. (2023) extensively explored the interplay between mitochondria dysfunction, cellular senescence, and SASP both in cultured cells and *in vivo*. To determine whether MOMP is a feature of cellular senescence, they observed a reduced co-localization of TOM20 (a mitochondrial membrane protein) and cytochrome c (Cyt c) in senescent human fibroblasts. Besides, the levels of cytosolic Cyt c and cleaved caspase-3 were elevated, along with the activation of BAX in senescent fibroblast strains (MRC5 and IMR90), further confirming MOMP occurs in cellular senescence as in apoptosis. Notably, this process of MOMP, a consequence of sublethal apoptotic stress, occurs only in a portion of mitochondria without inducing cell death. This phenomenon, termed as minority MOMP (miMOMP), occurs specifically during cellular senescence.

**FIGURE 1:**
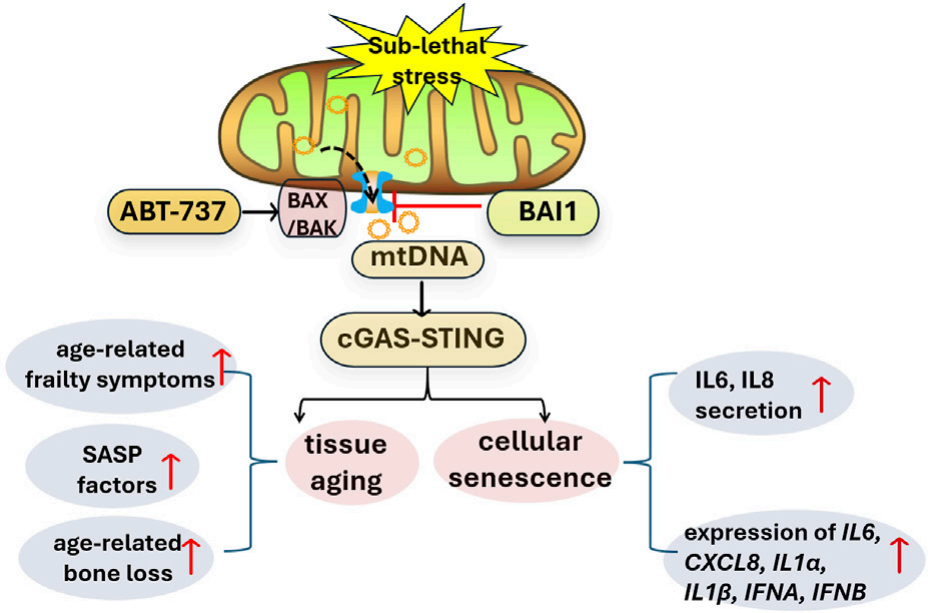
miMOMP activates mtDNA-cGAS-STING pathway and promotes cellular senescence and tissue aging. ABT-737 promoted BAX activation and formation of BAX/BAK macropore, thus induced miMOMP and mtDNA release, subsequently activated cGAS-STING pathway, inducing cellular senescence and tissues aging. BAI1, as a BAX inhibitor, blocked this signaling pathway. cGAS-STING: cyclic GMP-AMP synthase-stimulator of interferon genes; miMOMP: minority mitochondrial outer membrane permeabilization; mtDNA: mitochondria DNA; SASP: senescent associated secretory phenotype.

MOMP during apoptosis promotes the release of mtDNA into the cytoplasm, which in turn activates the cyclic GMP-AMP synthase-stimulator of interferon genes (cGAS-STING) pathway (McArthur et al., 2018). In line with this, Victorelli et al. (2023) observed an increased co-localization of mtDNA with mitochondrial transcription factor A (TFAM) in the cytoplasm of senescence models, which is considered as a preferred substrate of cGAS (Andreeva et al., 2017). Subsequently, they sought to investigate the correlation between miMOMP and SASP. Treatment low concentration of ABT-737, an inducer of miMOMP, significantly enhanced the secretion of SASP components, such as IL-6 and IL-8, and upregulated the expression of *IL6, CXCL8, IL1α, IL1β, IFNA* and *IFNB* in human fibroblasts, accompanied by an increased number of cytosolic mtDNA nucleoids. To examine the role of cGAS-STING pathway in SASP, Victorelli and colleagues demonstrated that the expression of cGAS-STING-induced interferon-stimulated genes (ISGs) was increased during senescence, which was dependent on mitochondria or mtDNA (Victorelli et al., 2023). Collectively, these findings confirm that miMOMP is responsible for SASP activation, which is mediated by mtDNA-cGAS-STING pathway in senescent cells (as shown in Figure 1).

An interesting issue is why miMOMP occurs in cellular senescence, that is, what drives MOMP limited to only a portion of mitochondria in senescent cells? Victorelli et al. (2023) found that senescent cells contain relatively few fragmented/fissioned mitochondria, which are the ones undergoing MOMP. Surprisingly, Cao et al. (2022) found mitochondrial fusion inhibits miMOMP by enabling homogeneous expression of anti-apoptotic BCL-2 proteins in mitochondrial. Meanwhile, mitochondrial fission facilitated miMOMP via accumulating pro-apoptotic BAX in U2OS and HeLa cells, indicating mitochondrial dynamics regulate miMOMP. Consistently, Victorelli et al. (2023) reported that disrupting mitochondrial fusion by knocking down the mitofusion-2 (*MFN-2*) enhanced BAX activation, mtDNA release, and SASP secretion. Alternatively, senescent cells exposure to mitochondrial uncoupler (carbonyl cyanide m-chlorophenyl hydrazine, CCCP), caused mitochondrial fragmentation and exacerbated mtDNA release as well as SASP production (Victorelli et al., 2023). Taken together, these findings underscore the critical role of mitochondrial dynamics in regulating miMOMP and highlight the potential of targeting mitochondrial fusion/fission pathways as a therapeutic strategy for modulating cellular senescence and its associated diseases.

Since accumulation of senescent cells is associated with numerous chronic diseases, thus far interests on safety and target engagement of senotherapy is rapidly growing. In their project, Victorelli and colleagues (Victorelli et al., 2023) have shown inhibiting miMOMP may be a novel target to counteract ageing and prolong health span. In cells, BAX is able to translocate to mitochondrial outer membrane and form oligomers to prompt MOMP. Treatment with BAX inhibitor BAI1 effectively suppressed BAX activation and inhibited MOMP in senescent MRC5 and IMR90 cells, resulting in a reduction of mtDNA release and a reduction of SASP factors secretion. Similarly, eltrombopag, another BAX inhibitor with a mechanism distinct from BAI1, also reduced several SASP factors in senescent MRC5 fibroblast, emphasizing the specific role of BAX in miMOMP-induced SASP. Furthermore, they found that BAI1 treatment alleviated the decline in neuromuscular coordination and delayed the development of age-related frailty symptoms in aged mice. Additionally, BAI1 treatment increased bone mass and strength, and improved bone microarchitecture. Importantly, BAI1 treatment effectively reduced the pro-inflammatory gene expression and decreased levels of circulating SASP factors in the bones and brains of old mice. Collectively, these findings highlight the potential of targeting miMOMP as a novel strategy for alleviating inflammation and mitigating the effects of cellular senescence, thus offering a promising approach to promote healthy aging and combat age-related diseases (Victorelli et al., 2023).

## Discussion

Currently, conventional intervention therapies including inhibition of anti-apoptotic regulators that confers senescent cell survival, inhibition of SASP (named senomorphics), and elimination of senescent cells via inducing apoptosis (named senolytics), along with immunological approaches for immune cell-mediated clearance of senescent cells, represent promising strategies to treat ageing and age-related diseases (Calabrò et al., 2024).

Current senolytic agents, such as dasatinib, quercetin, navitoclax, significantly eliminated senescent cells in aged mice by suppressing anti-apoptotic BCL-2 pathways (Lelarge et al., 2024). However, these agents also exhibited several limitations during clinical development, which has been extensively discussed in a recent literature (Calabrò et al., 2024). It is worth pointing out that cellular senescence plays a positive role in tumor suppression and the prevention of cellular transformation (Xiao et al., 2023). Given its dual nature, targeting senescence requires a delicate balance: selectively mitigating its harmful effects while preserving its beneficial characteristics remains a significant challenge. Strikingly, both deletion of *BAX/BAK* and BAI1 treatment did not alter the gene expression of the cyclin-dependent-kinase inhibitors, such as *Cdkn2a* (encoding p16^Ink4a^) and *Cdkn1a* (encoding p21) (Victorelli et al., 2023), indicating BAX inhibition only regulates the proinflammatory features of senescence without interfering with its tumor-suppressive functions. In addition, pharmacological inhibition of miMOMP (by BAX inhibitor BAI1) has the risk of off-target effects, however, genetic inhibition of miMOMP also inhibits the SASP *in vivo*. Importantly, as above mentioned, BAI1 treatment reduced inflammatory factors and improved various healthspan parameters, such as bone loss and frailty phenotypes during ageing, thereby achieving the goal of enriching the quality of life (Victorelli et al., 2023). Thus, miMOMP inhibition, which maintains mitochondrial function and specifically inhibiting SASP, may be a better therapeutic strategy for age-related diseases.

Many factors, including miMOMP, reactive oxygen species (ROS), epigenetic regulation, mammalian target of rapamycin (mTOR) and tumor suppressor pathways contribute to the induction of SASP and cellular senescence. Further investigations are needed to fully elucidate the mechanism and the crosstalk among these pathways that drive SASP. In addition, apart from BAI1, whether other small molecule allosteric inhibitors of BAX have a specific effect on inhibiting SASP in senescent cells (Garner et al., 2019) ? Smer-Barreto and colleagues recently conducted a computational screen of various chemical libraries and identified oleandrin, which demonstrated superior potency compared to other senotherapeutic agents, highlighting the potential of artificial intelligence (AI) (Smer-Barreto et al., 2023). Thus, AI may offer significant advantages on senotherapy, particularly by modeling the combination of different targets or different BAX inhibitors in the future.

## Conclusion

Taken together, miMOMP occurs during cell senescence and drives the production of SASP through activation of mtDNA-cGAS-STING pathway, which links cellular senescence to aging, providing a fresh perspective and insights into the therapeutic strategy aimed at delaying aging.

## Data Availability

The original contributions presented in the study are included in the article/supplementary material, further inquiries can be directed to the corresponding author.
